# Supplementary material for: Swiss public attitudes to human cryopreservation
Source: PLoS One. 2026 May 8;21(5):e0332005. doi: 10.1371/journal.pone.0332005 (PMC13155587; doi:10.1371/journal.pone.0332005)
Supplement: Attachment — Submitted filename: Response_to_Reviewers_auresp_2.docx [file pone.0332005.s003.docx]

Manuscript ID: PONE-D-25-45890

Title: Swiss Public Attitudes towards Cryopreservation

Dear Editor,

Thank you for the opportunity to revise our manuscript. We have addressed all comments from the reviewers and have adjusted the manuscript accordingly. Please find our point-by-point responses below.

Comments from the Academic Editor:

1. *Please ensure that your manuscript meets PLOS ONE's style requirements, including those for file naming.*
   Response: We have adjusted the formatting, including figure naming and author affiliations, to meet the guidelines.
2. *PLOS requires an ORCID iD for the corresponding author in Editorial Manager on papers submitted after December 6th, 2016.*
   Response: We have added the corresponding author’s ORCID ID in Editorial Manager.
3. *Please amend your list of authors on the manuscript to ensure that each author is linked to an affiliation. Authors’ affiliations should reflect the institution where the work was done.*
   Response: We have amended the authors list and affiliations.
4. *We note that there is identifying data in the Supporting Information file <S1 Complete Dataframe.xlsx>. Due to the inclusion of these potentially identifying data, we have removed this file from your file inventory. Prior to sharing human research participant data, authors should consult with an ethics committee to ensure data are shared in accordance with participant consent and all applicable local laws.*
   Response: We haven’t found any data that would compromise participant’s privacy (names, initials, specific dates, contact details, location, etc), therefore have not amended the S1 Complete Dataframe.xlsx file.
5. *Please include captions for your Supporting Information files at the end of your manuscript, and update any in-text citations to match accordingly.*
   Response: We have included a title and caption of the supplementary table at the end of the manuscript.
6. *If the reviewer comments include a recommendation to cite specific previously published works, please review and evaluate these publications to determine whether they are relevant and should be cited. There is no requirement to cite these works unless the editor has indicated otherwise.*
   Response: We have only included references deemed appropriate and relevant.
7. *On the previous request concerning identifying data, please remove the fields in the supporting data containing exact ages and retain only the age bins.*
   Response: Data containing exact ages have been removed from supporting data.
8. *Provide further detail in the main text on the consent process to demonstrate that informed consent was appropriately obtained from participants.*
   Response: We have added an ethics declaration statement in the Methodology section of the manuscript, detailing GDPR compliance and informed consent obtaining.
9. *Consistent with best practice and the WMA’s Declaration of Helsinki, I would reiterate the request for the study to be reviewed by an independent ethics panel, or provide clarification if such independent oversight has already taken place, in order to confirm that relevant ethical and legal requirements were followed (e.g. GDPR).*
   Response:
   This study is exempt from the full scope of Switzerland's Human Research Act (HRA, Art. 2), as it does not investigate human diseases, bodily functions, or public health outcomes, nor does it involve any form of physical or psychological intervention. The research consists exclusively of purely observational, anonymous surveys, which fall outside the HRA's regulatory remit. Data collection was conducted in full compliance with the Swiss Federal Act on Data Protection (FADP). We have added an ethics declaration to the manuscript and uploaded a merged document containing consent forms, terms & conditions and privacy policy from Positly alongside this revision.

Comments from Reviewer 1:

1. *My one main concern is regarding the conclusion here: "Survey results show that most participants were against making cryopreservation illegal (Fig 2). A total of 83.5% of respondents either opposed or remained neutral toward a ban, with 38.9% explicitly disagreeing or strongly disagreeing and 44.6% expressing neutrality. Only 16.4% agreed or strongly agreed with making it illegal."...*
   Response: Thank you for pointing this out. This has now been corrected.
2. *Also, some of the citations could be formatted better. For example, some of them seem to have authors, but the authors are not listed in the citation. It might also be useful to have a few citations in the beginning to the general topic of cryopreservation/biostasis/cryonics, so that readers know specifically what you are referring to.*
   Response: We have paid special attention to formatting the citations and added more of them to support our claims and reader’s understanding.
3. *One minor point regarding: "This suggests that some individuals may view cryopreservation as a way of coping with death anxiety". I would specifically describe this as a "problem-solving way of coping", rather than just a way of coping. Because theoretically, it's not just about feeling better, which is sometimes the connotation of the word "coping", but rather attempting to solve the problem of death.*
   Response: Reviewer’s minor point was taken into consideration, and we agree with the proposed idea that cryopreservation is a problem-solving venture. Our data suggests a significant correlation between fear of death and openness to cryopreservation, suggesting a view of cryopreservation way f coping with death anxiety, albeit a solution-oriented one.

Comments from Reviewer 2:

1. *Line 1: The title “Cryopreservation sentiment in Switzerland” is too broad. “Cryopreservation” covers embryos, gametes, organs, etc. The survey is specifically about human cryopreservation/cryonics. “Stated attitudes” would be more precise than “Sentiment”. My suggestion for an alternative title is “Swiss public attitudes to cryonics”.*
   Response: We have taken reviewer’s suggestions into consideration and adapted the title to: Swiss public attitudes to human cryopreservation.
2. *Line 8 and 34: The authors describe the 2022 EBF cryonics facility as the first in Europe. The authors should modify this claim, due to the KrioRus facility established in 2005 in geographical Europe. For instance, the authors might replace “Europe” with “Schengen Area”.*
   Response: Thank you for highlighting this. We have changed both lines from Europe to Schengen Area.
3. *Line 26: The authors should discuss and include references to at least some landmark publications on cryonics in bioethical journals, including Shaw Bioethics 2009, Moen JME 2015, Thau Bioethics 2020, as well as more recent article like Sauchelli Sci Eng Ethics 2024, Shao Int J Phil Stud 2025, German and Tretter Neuroethics 2025.*
   Response: We agree with this point and have expanded the Reference list with more publications in bioethical journals.
4. *Figure 1: The authors might consider representing this data as a pie chart or a diverging stacked horizontal bar chart.*
   Response: we find that the simplicity of representations catches the readers eye and have not modified the figure.
5. *Line 128: The authors may also relate their findings regarding family wishes to Hillenbrink R, Wareham CS. Mourning the frozen: considering the relational implications of cryonics. J Med Ethics. 2024*
   Response: we briefly discussed the mentioned publication in the “Openness to Cryopreservation as Philosophical Stance” section of the Discussion (Page 16, lines 315 – 317).
6. *Line 254 and Line 277: If successful, cryonics could have pronounced benefits. It is therefore of general interest to elucidate the underlying psychological and sociological causes for its appeal to the general population in different framings. The manuscript provides some engagement with this topic by pointing to Bandura's reciprocal determinism, but the authors should extend their discussion of potential causal effects.*
   Response: We have expanded our Openness–Awareness Dynamic discussion section (Page 13, lines 301-304) and Openness to Cryopreservation as Philosophical Stance section (Page 15, lines 334-335), to include potential causal effects.
7. *Please clarify competing interests, including affiliations and equity stakes related to Tomorrow Biostasis, the European Biostasis Foundation, and other pertinent entities discussed in the manuscript.*
   Response: We have added a section on competing interests.

Sincerely,

Emil Kendziorra

On behalf of all authors.
